# Implementation science and stigma reduction interventions in low- and middle-income countries: a systematic review

**DOI:** 10.1186/s12916-018-1237-x

**Published:** 2019-02-15

**Authors:** Christopher G. Kemp, Brooke A. Jarrett, Churl-Su Kwon, Lanxin Song, Nathalie Jetté, Jaime C. Sapag, Judith Bass, Laura Murray, Deepa Rao, Stefan Baral

**Affiliations:** 1 Department of Global Health, University of Washington, Seattle, WA USA; 2 Department of Epidemiology, Johns Hopkins University, Baltimore, MD USA; 3 Department of Neurology, Icahn School of Medicine at Mount Sinai, New York City, NY USA; 4 Departments of Public Health and Family Medicine, School of Medicine, Faculty of Medicine, Pontificia Universidad Católica de Chile, Santiago, Chile; 5 Dalla Lana School of Public Health, University of Toronto, Toronto, ON Canada; 6 Office of Transformative Global Health, Institute for Mental Health Policy Research, Centre for Addiction and Mental Health, Toronto, ON Canada; 7 Department of Mental Health, Johns Hopkins University, Baltimore, MD USA

**Keywords:** Stigma, intervention, implementation science, systematic review, low- and middle-income countries

## Abstract

**Background:**

Interventions to alleviate stigma are demonstrating effectiveness across a range of conditions, though few move beyond the pilot phase, especially in low- and middle-income countries (LMICs). Implementation science offers tools to study complex interventions, understand barriers to implementation, and generate evidence of affordability, scalability, and sustainability. Such evidence could be used to convince policy-makers and donors to invest in implementation. However, the utility of implementation research depends on its rigor and replicability. Our objectives were to systematically review implementation studies of health-related stigma reduction interventions in LMICs and critically assess the reporting of implementation outcomes and intervention descriptions.

**Methods:**

PubMed, CINAHL, PsycINFO, and EMBASE were searched for evaluations of stigma reduction interventions in LMICs reporting at least one implementation outcome. Study- and intervention-level characteristics were abstracted. The quality of reporting of implementation outcomes was assessed using a five-item rubric, and the comprehensiveness of intervention description and specification was assessed using the 12-item Template for Intervention Description and Replication (TIDieR).

**Results:**

A total of 35 eligible studies published between 2003 and 2017 were identified; of these, 20 (57%) used qualitative methods, 32 (91%) were type 1 hybrid effectiveness-implementation studies, and 29 (83%) were evaluations of once-off or pilot implementations. No studies adopted a formal theoretical framework for implementation research. Acceptability (20, 57%) and feasibility (14, 40%) were the most frequently reported implementation outcomes. The quality of reporting of implementation outcomes was low. The 35 studies evaluated 29 different interventions, of which 18 (62%) were implemented across sub-Saharan Africa, 20 (69%) focused on stigma related to HIV/AIDS, and 28 (97%) used information or education to reduce stigma. Intervention specification and description was uneven.

**Conclusion:**

Implementation science could support the dissemination of stigma reduction interventions in LMICs, though usage to date has been limited. Theoretical frameworks and validated measures have not been used, key implementation outcomes like cost and sustainability have rarely been assessed, and intervention processes have not been presented in detail. Adapted frameworks, new measures, and increased LMIC-based implementation research capacity could promote the rigor of future stigma implementation research, helping the field deliver on the promise of stigma reduction interventions worldwide.

**Electronic supplementary material:**

The online version of this article (10.1186/s12916-018-1237-x) contains supplementary material, which is available to authorized users.

## Background

Health-related stigma – the co-occurrence of labeling, stereotyping, separating, status loss, and discrimination associated with a specific disease in the context of power imbalance [1] – deepens health disparities and drives population mortality and morbidity [2]. Interventions to alleviate stigma and its consequences are demonstrating effectiveness across a range of conditions, including HIV/AIDS, mental and substance use disorders, leprosy, epilepsy, and tuberculosis [3–10]. For example, social contact interventions, which facilitate interactions between individuals with a stigmatizing condition and those without it, have been shown to be effective at reducing community stigmatizing beliefs about mental health [6]; individual- and group-based psychotherapeutic interventions have been shown to reduce internalized stigma associated with HIV and mental health conditions [3, 10]; and socioeconomic rehabilitation programs have been shown to reduce stigmatizing attitudes towards people with leprosy [5]. Observed effects have tended to be small-to-moderate and limited to changes in attitudes and knowledge, with less evidence concerning long-term impacts on behavior change and health [11, 12]. Stigma can be intersectional, wherein multiple stigmatizing identities converge within individuals or groups, and effective interventions often grow complex to reflect this reality [13]. Interventions may be multi-component and multi-level [3], meaning that they may be especially difficult to implement, replicate, and disseminate to new contexts [14].

Few stigma reduction interventions move beyond the pilot phase of implementation, and those that do have tended to be in high-income countries. For example, mass media campaigns to reduce the stigma associated with mental health have been implemented at scale and sustained over time in the England, Scotland, Canada, New Zealand, and Australia [11]; however, most interventions do not reach those who need them. This is especially true in low- and middle-income countries (LMICs), where reduced access to resources and lack of political support for stigma reduction interventions compound the burden and consequences of stigma [15, 16]. For example, most LMICs spend far less than needed on the provision of mental health services [17], making large-scale investment in mental health stigma reduction programs unlikely without strong evidence of affordability and sustainability. Furthermore, stigma in low-resource settings tends to be a greater impediment to accessing services than elsewhere [18]. Anti-homosexuality laws and other legislation criminalizing stigmatized identities both increase the burden of stigma and prevent the implementation of effective services and interventions [19]. The same cultural and structural factors that drive and facilitate stigmatizing attitudes threaten the credibility and uptake of the interventions themselves [20].

**Fig. 1:**
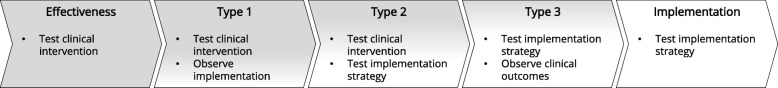
Continuum of study designs from effectiveness to implementation. As defined by Curran et al. [31]

Implementation science seeks to improve population health by leveraging interdisciplinary methods to promote the uptake and dissemination of effective, under-used interventions in the real world [21]. The emphasis is on implementation strategies, namely on approaches to facilitate, strengthen, or sustain the delivery of evidence-based technologies, practices, and services [22, 23]. Implementation science studies use qualitative and quantitative methods to measure implementation outcomes, including acceptability, adoption, appropriateness, cost, feasibility, fidelity, penetration, and sustainability (Table 1) [24]; these are indicators of implementation success and process, proximal to service delivery and patient health outcomes. Increasingly, studies use psychometrically validated measures of implementation outcomes [25, 26]. A range of theoretical frameworks support implementation science, including those that can be used to guide the translation of research into practice (e.g., the Canadian Institutes of Health Research Model of Knowledge Translation [27]), study the determinants of implementation success (e.g., the Consolidated Framework for Implementation Research [28]), and evaluate the impact of implementation (e.g., the RE-AIM framework [29]) [30]. Depending on the level of evidence required and the research questions involved, studies fall along a continuum from effectiveness, to hybrid effectiveness-implementation [31], to implementation (Fig. 1). Whereas effectiveness studies focus a priori on generalizability and test the effect of interventions on clinical outcomes [32], hybrid study designs can be used to test intervention effects while examining the process of implementation (type 1), simultaneously test clinical interventions and assess the feasibility or utility of implementation interventions or strategies (type 2), or test implementation interventions or strategies while observing clinical outcomes (type 3) [31]. Non-hybrid implementation studies focus a priori on the adoption or uptake of clinical interventions in the real world [33].

**Table 1:** Implementation outcome definitions

Implementation Outcome	Definition^a^
Acceptability	Perception that the intervention is agreeable, satisfactory, or confers relative advantage
Adoption	Early uptake or intent to try
Appropriateness	Pre-adoption perception of practicability, fit, or relevance
Cost	Marginal cost, cost-effectiveness, cost-benefit
Feasibility	Whether the intervention is suitable for everyday use, practicable, or fits with provider workflow
Fidelity	Whether the core components of an intervention were implemented as intended
Penetration	Spread within an eligible population or level of institutionalization
Sustainability	Extent to which an intervention can be maintained, routinized, or institutionalized by a provider or facility

^a^As defined by Proctor et al. (2011) [24]

Implementation science has particular relevance to the goal of delivering effective stigma reduction interventions in LMICs, offering tools to identify, explain, and circumvent barriers to implementation given severe resource constraints [34]. It can be used to study and improve complex interventions whose multiple, interacting components blur the boundaries between intervention, context, and implementation [14] and has the potential to generate evidence of affordability, scalability, and sustainability, which could be used to convince policy-makers and donors to invest in future implementation [35]. Moreover, it could bring policy-makers, providers, patients, and other stakeholders into the research process, promoting engagement around the study and delivery of interventions that may themselves be stigmatized [36]. However, the utility of implementation research depends on its rigor and replicability. To encourage growth and strength in the field of stigma implementation research, it is important to summarize previous work in the area, evaluate that rigor and replicability, and articulate priorities for future research. Our objectives were to systematically review implementation studies of health-related stigma reduction interventions in LMICs and critically assess the reporting of implementation outcomes and intervention descriptions.

## Methods

We registered our systematic review protocol in the International Prospective Register of Systematic Reviews (PROSPERO #CRD42018085786) and followed the Preferred Reporting Items for Systematic Reviews and Meta-Analyses (PRISMA) guidelines [37].

### Search strategy

One author (CK) searched four electronic bibliographic databases (PubMed, CINAHL, PsycINFO, and EMBASE) through November 15, 2017, for studies fulfilling four search concepts – stigma, intervention, implementation outcomes, and LMICs. We developed a list of terms for each concept in collaboration with an information scientist. The full search strategy for all databases is presented in Additional file 1. The PsycINFO search excluded dissertations, while the CINAHL search was restricted to academic journals. Finally, the reference lists of included studies were reviewed for additional publications.

### Study selection

Studies were included in any language that (1) collected empiric data, (2) evaluated implementation of an intervention whose primary objective was to reduce stigma related to a health condition, (3) were based in a LMIC according to the World Bank [38], and (4) reported at least one implementation outcome as defined by Proctor et al. [24]. Studies evaluating interventions targeting stigma related to marginalized identities, behaviors, beliefs, or experiences (e.g., stigma related to race, economic status, employment, or sexual preference) were excluded if the interventions did not also target stigma related to a health condition. Unpublished and non-peer-reviewed research were excluded. Qualitative and quantitative studies had the same inclusion and exclusion criteria. The Covidence tool was used to remove duplicate studies and to conduct study screening [39]. A mix of two authors from a team of four (CK, BJ, CSK, and LS) independently screened all titles, abstracts, and full-text articles, and noted reasons for excluding studies during full-text review. Studies passed the title/abstract screening stage if the title or abstract mentioned stigma reduction and if it was possible that the study had been conducted in a LMIC. Studies passed the full-text screening stage if all criteria above were met. Disagreements were resolved through discussion until consensus was reached.

### Data abstraction

Two authors (CK and BJ) independently piloted a structured abstraction form with two studies; all co-authors reviewed, critiqued, and approved the form. For each study, one of three authors (CK, BJ, and CSK) abstracted study and intervention characteristics (Table 2) onto a shared spreadsheet. One of the two remaining authors verified each abstraction, and the group of three resolved any disagreement through discussion.

**Table 2:** Study and intervention characteristics

Level	Description	**Reference**
Study		
Year of publication	—	—
Implementation frameworks used	e.g., RE-AIM [29] or Consolidated Framework for Implementation Research [28]	Nilsen [30]
Study design	Qualitative, cross-sectional, cohort, non-randomized pre/post with and without controls, individual and cluster randomized trials, economic evaluations, or other	—
Study type	Effectiveness, type 1 hybrid, type 2 hybrid, type 3 hybrid, implementation, or scale-up	Curran et al. [31]
Study population	Community, patient, provider, and/or policy-makers	—
Implementation outcomes reported	Acceptability, adoption, appropriateness, feasibility, fidelity, cost, penetration, and/or sustainability	Proctor et al. [24]
Non-implementation outcomes reported	Outcomes related to stigma, service delivery, and patient health	—
Key findings	—	—
Reporting of implementation outcomes		—
Included in study objectives?	Whether the implementation outcome(s) were included in the study’s prespecified objectives	
Hypothesis or conceptual model stated?	Whether the implementation outcomes were motivated by a hypothesis or conceptual model	
Methods for outcomes specified?	Whether the methods for measuring the implementation outcomes were included	
Used validated measure(s)?	Whether the measures used were from or based on a validated measure	
Sample size specified?	Whether the implementation outcomes included the sample size of the population assessed	
Intervention		
Intervention description	—	—
WHO Region	Sub-Saharan Africa, East Asia and Pacific, South Asia, Middle East and North Africa, Europe and Central Asia, Latin America and the Caribbean	—
Stigmatizing health condition	—	—
Type of stigma reduction intervention	Information/education, skill development, counseling or support, contact events, structural, biomedical	Stangl et al. [3]
Stigma domain targeted	Driver, facilitator, and/or manifestation	Stangl et al. [3]
Type of stigma targeted	Community, experienced, internalized, anticipated, and/or unclear	Turan et al. [40]
Intervention specification using the TIDieR Checklist		Hoffman et al. [41]
Why	Intervention motivated with a rationale, theory, or goal	
What	Description or link of the intervention’s physical or informational materials	
Who provided	Expertise, background, and any specific training of the person implementing the intervention	
How	Mode of delivery (e.g., face-to-face)	
Where	Type of location of the intervention	
When and how much	Timing, duration, dose, and intensity of the intervention	
Tailoring	Intervention is personalized to participants or groups of participants	
Modifications	Whether the intervention was modified during the course of the study	
How well was fidelity assessed	Methods for assessing fidelity	
Quality of fidelity	If fidelity was assessed, the rating of the fidelity	

At the study level, we collected research questions, methods and study types, implementation research frameworks used, years of data collection, study populations, implementation outcomes reported [24], stigma, service delivery, patient health, and/or other outcomes reported, study limitations, and conclusions or lessons learned. Studies were categorized as effectiveness, type 1, 2, or 3 hybrid effectiveness-implementation [31], or implementation, according to Curran et al. [31]. We noted the stage of intervention implementation at the time of each study as either pilot/once-off, scaling up, implemented and sustained at scale, or undergoing de-implementation. Studies were considered to have used an implementation research framework if authors specified one within the introduction or methods. Implementation outcomes were defined according to Proctor et al. [24]. Patient-level service penetration – the percent of eligible patients receiving an intervention – was considered a form of penetration, though this distinction is not clear in Proctor et al. [24]. We developed a five-item rubric to assess the quality of reporting of implementation outcomes, noting whether the authors included the implementation outcomes in their study objectives; whether they specified any hypotheses or conceptual models for the implementation outcomes; whether they described measurement methods for the implementation outcomes; whether they used validated measures for the implementation outcomes [25]; and whether they reported the sample sizes for the implementation outcomes.

At the intervention level, we collected intervention names, intervention descriptions, countries, associated stigmatizing health conditions, and target populations. Interventions were categorized based on type, including information/education, skills, counselling/support, contact, structural, and/or biomedical [3]; socio-ecological level, including individual, interpersonal, organizational, community, and/or public policy; stigma domain targeted, including driver, facilitator, and/or manifestation [3]; and finally the type of stigma targeted, including experienced, community, anticipated, and/or internalized [40]. The 12-item Template for Intervention Description and Replication (TIDieR) was used to evaluate the comprehensiveness of intervention description and specification by the studies in the sample [31]. TIDieR is an extension of item five of the Consolidated Standards of Reporting Trials (CONSORT), providing granular instructions for the description of interventions to ensure sufficient detail for replicability [41]. Implementation science journals encourage the use of TIDieR or other standards when describing interventions [42]. Each item in the TIDieR checklist (e.g., who provides the intervention? What materials are used?) was counted as present if any aspect of the item was mentioned, regardless of quality or level of detail. When multiple studies in the sample evaluated the same intervention, TIDieR intervention specification was assessed across the studies. Risk of bias was not assessed, as the goal was not to synthesize results across the studies in the sample.

### Analysis

We calculated percentages for categorical variables and means and standard deviations (SD) for continuous variables. An implementation outcome reporting score was calculated for each study by summing the number of rubric items present and dividing by the total number of applicable items. A TIDieR specification score out of 12 was calculated for each intervention by summing the number of checklist items reported across studies of the same intervention and dividing by the total number of applicable items. These variables were used to summarize the aims, methods, and results of the studies and interventions in the sample. Qualitative synthesis and quantitative meta-analysis of study findings was not possible, given the heterogeneity in research questions and outcomes.

## Results

### Study selection

**Fig. 2:**
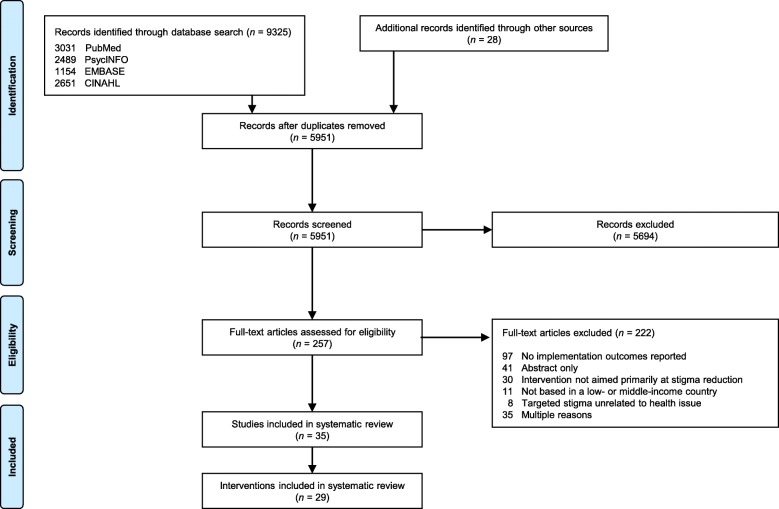
PRISMA flow diagram

We screened 5951 studies and assessed 257 full-text articles for eligibility. A total of 35 studies met all eligibility criteria (Fig. 2) [43–77] and evaluated 29 different stigma reduction interventions (Table 3).

**Table 3:** Included studies (*n* = 35) and associated interventions (*n* = 29) by year of publication

Study										Intervention				
First Author Citation	Year	Design	Study Type	Imp. Stage	Study Population	Imp. Outcome(s)	Imp. Outcome Score	Other Outcome(s)	Key Findings	Description	Name	Country	Stigmatizing Condition	TIDieR Score
el-Setouhy [43]	2003	CS; PPwoC	Type 1	Pi	Co	Ac	40%	S	Children liked the comic book and its contents and found this book easy to understand	Comic book for Egyptian schoolchildren	n/s	Egypt	LF	55%
Norr [44]	2004	Qual; PPwC	Eff	Pi	Co	Su	0%	S O	Leaders continued to offer intervention post-funding	Peer group intervention	n/s	Botswana	HIV; STDs	64%
Witter [45]	2004	Qual; Econ	Type 1	Sc	Co Pa	Ac Ad C Fe Su	20%	S H O	Acceptable and effective tool with low costs, though participants still cannot bear cost; hard to keep momentum and extend coverage	Book of family background, memories, hopes	Memory Book	Uganda	HIV	55%
Lawoyin [46]	2007	Qual; PPwoC	Type 1	Pi	Co	Ac	20%	S	Satisfaction with content and appreciation for social support	Life skills education	n/s	Nigeria	HIV	64%
Boulay [47]	2008	CS	Type 1	Sc	Co	Pe	50%	S	51% of men and 53% of women recalled hearing or seeing the slogan; broadcast media reached the greatest number of men and women	Mass media, promotional materials, training for local religious leaders	Reach Out, Show Compassion	Ghana	HIV	55%
Finkelstein [48]	2008	Qual; iRCT	Type 1	Pi	Co	Ac Fe	40%	S	Students liked how program worked; none had problems operating the program Content, user interface, and learning process could be improved	Anti-stigma computer program and print materials	Computer Assisted Education system	Russia	MH	36%
Khumalo-Sakutukwa [49]	2008	cRCT; QI/QA	Type 1	Pi	Co Pr	Fi Pe	40%	SD	4-fold increase in testing in intervention vs. control communities; 95% adherence to intervention	Community mobilization, community HIV voluntary counselling and testing, and post-test support services	Project Accept	Tanzania, Zimbabwe, South Africa, Thailand	HIV	91%
Lapinski [50]	2008	Qual; CS; iRCT	Type 1	Pi	Co	Ac Ap	40%	S SD	Enjoyment was high and men and women did not differ in their enjoyment of the film or perception of credibility	Film	Starting Over	Nigeria	HIV	55%
Pappas-DeLuca [51]	2008	CS	Type 1	Sc	Co	Pe	75%	S SD O	Nearly one half reported listening each week, 15% for 1 year or longer, and 19% talking to someone about Makgabaneng in past 3 months	Serial radio drama	Makgabaneng	Botswana	HIV	64%
Zeelen [52]	2010	Qual	Imp	Pi	Pa Pr	Ac Fe	25%		Storytelling seen as useful; some listeners distracted by other people; some had to see provider and missed story message	Clinic waiting room storytelling	n/s	South Africa	HIV	55%
Gurnani [53]	2011	M&E	Imp	Sc	Co Po	Pe	50%	SD O	Increase in FSW population reached from 40% to 85%; 61.5% of police force received training; crisis management teams supported 92% of rights violations in redress	Advocacy, legal empowerment, police and media sensitization	n/s	India	HIV	45%
Watt [54]	2011	Qual	Type 1	Pi	Co Pa	Ac Fi	60%	S SD H O	Patients judged usefulness 4.76 out of 5; intervention was delivered as intended in the manual – all 20 sectionscovered in each session	Support group for antiretroviral therapy patients	Zinduka	Tanzania	HIV	64%
Denison [55]	2012	CS; Econ	Type 1	Sc	Co	C	60%	S O	Total annual cost of US $501,516; average annual cost per school of US $8222; Adolescent Sexual and Reproductive Health Life Skills Education pillar had highest cost (US $2197)	Volunteer peer educators, youth resource center, extracurricular activities, community events, teacher training	School HIV/AIDS Education Program	Zambia	HIV	64%
Rice [56]	2012	cRCT	Type 1	Pi	Co	Pe	100%	S O	Rise in communication about intervention messages in the intervention Chinese markets was at first moderate and then substantial	Training market vendors, community-popular opinion leaders	n/s	China	HIV; STDs	64%
Al-Iryani [57]	2013	Qual	Type 1	Pi	Co Pr Po	Ac Fe	60%	SD	76.6% of students considered intervention beneficial; program was acceptable because it was framed as ‘life skills’, did not take away from academics, and was supported by school staff	School peer education program	n/s	Yemen	HIV	73%
Catalani [58]	2013	Qual; PPwoC	Type 1	Pi	Co	Ac	75%	S	Storyline seemed believable and possible; participants responded positively to each medium	Film	Prarambha	India	HIV	64%
Li [59]	2013	cRCT	Type 1	Pi	Pr	Ac	20%		Approach accepted by providers; messages were relevant to provider self-interests	Training providers as popular opinion leaders	n/s	China	HIV	73%
Li [60]	2013	cRCT	Type 1	Pi	Pr	Fe	0%	S	Intervention appropriately covered key issues relevant to provider daily practice					
Murray [61]	2013	Qual; PPwoC	Type 1	Pi	Co Pa Pr Po	Ap	100%	SD H	Preference for treatment that serves both children and adolescents; treatment that addressed traumatic grief; cross-cultural appropriateness and/or flexibility to adapt; and evidence of effectiveness	Psychosocial therapy for orphans and vulnerable children	Trauma-Focused Cognitive Behavioral Therapy	Zambia	MH	100%
Murray [62]	2013	QI/QA; PPwoC	Type 1	Pi	Pa	Fi	40%	S SD H	Treatment conducted with fidelity due to close monitoring built into Apprenticeship model					
Murray [63]	2014	Qual	Type 1	Pi	Co Pa Pr	Ac Fe	75%		Counselors liked structure and flexibility, reported positive changes in clients, and discussed cultural adaptation around activities and language; children and caregivers stated positive changes attributed to trauma-focused cognitive behavioral therapy					
French [64]	2014	Qual	Type 1	Pi	Co Pa	Ac	75%	S	Taking on projects was difficult but empowering	Training people living with HIV and peers to conduct community projects	n/s	South Africa	HIV	55%
French [65]	2015	Qual	Type 1	Pi	Co Pa	Ac Fe	20%	S	Some struggled with their projects; many found projects challenging and exhausting, yet interesting and exciting					
Shah [66]	2014	PPwC	Type 1	Pi	Co	Ac Fe	60%	S	Both sessions rated highly; few participants said materials made them uncomfortable; most felt that they could be honest about their beliefs and behaviors	Stigma reduction curriculum for nurses	n/s	India	HIV	64%
Lusli [67]	2015	Qual	Type 1	Pi	Pr	Ap Fe	50%	SD	Effective communication skills were important; physically disabled counsellors had challenges making contact and providing counselling; distance between clients was challenging	Rights-based counselling of people with leprosy by peer and lay counsellors	Stigma Assessment and Reduction of Impact: Counselling	Indonesia	Lepr.	73%
Lusli [68]	2016	Qual; cRCT	Type 1	Pi	Co Pa	Ac Fe	50%	S SD O	Participants appreciated counselling; counsellors needed intense supervision; not easy to manage client conditions and characteristics; peer counselors were preferred; family counselling preferred overall, but integrating family, individual, and group counselling was best approach					
Peters [69]	2015	Qual; cRCT	Type 1	Pi	Co	Fe	0%	S SD	Challenges related to convincing key of value of contact event, logistics (weak audio system, inappropriate venue, too many people and limited time), audience (tired, less involved), and Stigma Assessment and Reduction of Impact team (cancelations, delayed)	Contact events using testimonies, participatory videos, and comics	Stigma Assessment and Reduction of Impact: Contact and Video	Indonesia	Lepr.	64%
Peters [70]	2016	Qual	Type 1	Pi	Pa	Ac Ap Fe	25%	S	Support of video process; some participants had physical limitations and needed additional technical training in videography					
Salmen [71]	2015	Qual	Type 1	Pi	Co Pa	Ac	50%	S O	Participants found testimonials and role-modeling to be encouraging and gave them courage to disclose, though some concerns about group testing and disclosure process were expressed	Microclinic of neighbors, relatives, and friends trained to provide psychosocial, nutritional, and adherence support	n/s	Kenya	HIV	64%
Figueroa [72]	2016	CS	Type 1	Sc	Co	Pe	50%	S O	Almost two-thirds (63%) of the control group said that they had heard of TT; exposure to TT was higher among those in the intervention group	Facilitated community dialogues and radio magazines	Tchova Tchova Histórias de Vida: Diálogos Comunitários	Mozambique	HIV	55%
Tekle-Haimanot [73]	2016	Qual; PPwoC	Type 1	Pi	Pr	Ap	60%	S	Providers suggested comic be distributed among school children and felt it was easy to read and understand, well presented and illustrated for a non-medical person	Comic book for Ethiopian schoolchildren	We’ll Make It	Ethiopia	Epil.	55%
Tora [74]	2016	Qual	Type 1	Pi	Co Pa	Fi	0%	S O	Weaknesses observed during training and household education sessions; some educators provided superficial presentations, improper use of supplementary examples, and incomplete messages	Lay health educator-delivered educational modules and booster sessions	Inherited susceptibility education module	Ethiopia	Pod.	82%
Wilson [75]	2016	Qual; PPwoC	Type 1	Pi	Co Pa Pr	Ac Fe	50%	H O	DVD players did not have high volume; few electrical outlets; participants identified with the Salvadorian patients and understood video content; short duration of video not disruptive to TB clinic schedules	Educational video	n/s	El Salvador	TB	64%
Lyons [76]	2017	CS; Coh	Type 1	Pi	Co	Ac Fe	40%	S H	63.9% of MSM and 82.5% of FSW agreed that the workshops were effective in addressing stigma; 68.0% of MSM and 81.6% of FSW self-reported that the workshops helped them think about how to cope with stigma	Peer-led group sessions, training health workers, and web-based referral system	n/s	Senegal	HIV	64%
Oduguwa [77]	2017	PPwC	Type 1	Pi	Co	Ac	40%	O	Most participants liked the program because it increased their awareness about mental illness; some noted that hearing about mental illness created fear; majority affirmed that the program benefited them, the school, and the family	Mental health awareness training	n/s	Nigeria	MH	64%

Designs: *CS* cross-sectional, *Coh* cohort, *M&E* monitoring and evaluation, *PPwC* non-randomized pre/post study with control, *PPwoC* non-randomized pre/post study without control*, iRCT* individual randomized controlled trial*, cRCT* cluster randomized controlled trial, *Qual* qualitative, *QI/QA* quality improvement/quality assurance

Study types: *Eff* effectiveness, *Type 1* type 1 hybrid effectiveness-implementation, *Imp* implementation

Implementation stages: *Pi* pilot/once-off implementation, *Sc* sustained implementation at scale

Study populations: *Co* community members, *Pa* patients, *Pr* providers, *Po* policy-makers

Implementation outcomes: *Ac* acceptability, *Ap* appropriateness, *Ad* adoption, *C* costs, *Fe* feasibility, *Fi* fidelity, *P* penetration, *S* sustainability

Other outcomes: *S* stigma, *SD* service delivery, *H* patient health, *O* other outcome

Stigmatizing conditions: *Epil* epilepsy, *Lepr* leprosy, *LF* lymphatic filariasis, *MH* mental health, *Pod* podoconiosis, *STD* sexually transmitted disease, *TB* tuberculosis

*FSW* female sex worker, *MSM* men who have sex with men, *n/s* not specified, *TT* Tchova Tchova Histórias de Vida

### Study characteristics

The 35 studies in the sample were published between 2003 and 2017; the median year of publication was 2013 (Table 4). Study designs varied and included both qualitative and quantitative methods; 20 (57%) adopted at least one qualitative method, including interviewing, focus groups, or observation, while 8 (23%) reported results from cross-sectional surveys. One was an effectiveness study, with no a priori intent to assess implementation outcomes. The majority (32, 91%) were type 1 hybrid effectiveness-implementation studies; for example, Shah et al. [66] paired an effectiveness study with a process evaluation in order to assess provider-level acceptability and feasibility. None were type 2 or type 3 hybrid studies. Two were implementation studies; for example, Gurnani et al. [53] used routinely collected monitoring and evaluation data to assess the penetration of a structural intervention to reduce stigma around HIV/AIDS and sex work. Most (29, 83%) were evaluations of once-off or pilot implementations, while 6 (17%) evaluated implementation at scale. None evaluated the interventions undergoing scale-up, and none evaluated the process of de-implementation. No studies adopted a formal theoretical framework for implementation research.

**Table 4:** Study-level descriptive statistics (*n* = 35)

	Total (%)
Median publication year (range)	2013 (2003–2017)
Implementation research framework	0 (0%)
Study design^a^	
Qualitative	20 (57%)
Cross-sectional	8 (23%)
Cohort	1 (3%)
Non-randomized pre/post without control	7 (20%)
Non-randomized pre/post with control	3 (9%)
Individual randomized controlled trial	2 (6%)
Cluster randomized controlled trial	6 (17%)
Policy analysis	0 (0%)
Economic evaluation	2 (6%)
Other	3 (9%)
Study type	
Effectiveness	1 (3%)
Type 1 hybrid	32 (91%)
Type 2 hybrid	0 (0%)
Type 3 hybrid	0 (0%)
Implementation	2 (6%)
Implementation stage	
Pilot/once-off	29 (83%)
Scaling up	0 (0%)
Implemented and sustained at scale	6 (17%)
De-implementation	0 (0%)
Study population^a^	
Community	28 (80%)
Patients	13 (37%)
Providers	10 (29%)
Policy-makers	3 (9%)
Implementation outcomes reported^a^	
Acceptability	20 (57%)
Adoption	1 (3%)
Appropriateness	5 (14%)
Cost	2 (6%)
Feasibility	14 (40%)
Fidelity	4 (11%)
Penetration	6 (17%)
Sustainability	2 (6%)
Implementation outcome reporting^a^	
Mean reporting score (SD)	40% (30%)
Included in study objectives?	14 (40%)
Hypothesis or conceptual model stated?	3 (9%)
Methods for outcomes specified?	28 (80%)
Used validated measure(s)?	0 (0%)
Sample size specified?	24 (69%)
Other outcomes reported^a^	
Stigma	25 (71%)
Service delivery	12 (34%)
Patient health	7 (20%)
Other	13 (37%)

^a^≥1 response per study possible

Patient, provider, or community-level acceptability (20, 57%) and feasibility (14, 40%) were the most frequently reported implementation outcomes. Though authors usually reported whether participants found activities useful, enjoyable, or difficult, they rarely described why. Penetration was also relatively common (6, 17%). In comparison, appropriateness and fidelity were reported in 5 (14%) and 4 (11%) studies, respectively, while cost and sustainability were reported twice each, and adoption was reported once. In addition to these implementation outcomes, stigma (25, 71%) and service delivery outcomes (12, 34%) were most frequently reported, while patient health outcomes were rarely assessed (7, 20%).

Implementation outcome reporting scores were low, with a mean of 40% (SD 30%); 14 (40%) studies mentioned implementation outcomes in their study objectives, while 3 (9%) prespecified a hypothesis or conceptual model to explain implementation outcomes. For example, Rice et al. [56] used diffusion of innovation theory to inform their hypothesis about the penetration of messaging in intervention settings. Though 28 (80%) studies described methods for collecting implementation outcomes and 24 (69%) documented a sample size for those outcomes, none used validated measures of implementation outcomes in their quantitative data collection.

### Intervention characteristics

Of the 29 interventions in the sample, 18 (62%) were implemented in sub-Saharan Africa (Table 5), 20 (69%) focused on stigma related to HIV/AIDS, and fewer addressed mental health (3, 10%), leprosy (2, 7%), or other conditions (6, 21%); the majority (28, 97%) used information or education to reduce stigma. For example, the *Tchova Tchova* program in Mozambique broadcasted HIV education over the radio, including a debate segment where listeners could ask questions to an HIV specialist [72]. Skill- and capacity-building were the next most common types of stigma reduction interventions (13, 45%), followed by counseling (6, 21%) and contact events (6, 21%). The Stigma Assessment and Reduction of Impact program in Indonesia, for instance, taught participatory video production skills to people affected by leprosy [67, 68], while the Trauma-Focused Cognitive Behavioral Therapy program in Zambia counseled orphans and vulnerable children to reduce shame-related feelings around sexual abuse [61–63]. Few interventions used structural (1, 3%) or biomedical (1, 3%) approaches to reduce stigma. The drivers of stigma were targeted by 28 (97%) studies, while few targeted its facilitators (4, 14%) or manifestations (10, 34%). In Senegal, the HIV Prevention 2.0 study targeted all three through its Integrated Stigma Mitigation Intervention approach, wherein drivers related to knowledge and competency of service providers, facilitators related to peer support and peer-to-peer referral, and manifestations related to individual self-stigma and self-esteem [76]. Most interventions (24, 83%) focused on reducing community stigma, while fewer targeted experienced (11, 38%), anticipated (7, 24%), or internalized stigma (9, 31%). For example, the Indian film *Prarambha* was produced to raise awareness about HIV and designed to be viewed by individuals in HIV-vulnerable communities, thus targeting a driver of community stigma related to HIV [58]. While many interventions operated at the individual (23, 79%) and interpersonal levels (14, 48%), fewer were implemented at the community (11, 38%), organizational (6, 21%), or public policy (1, 3%) levels. Several interventions at the community, organizational, or public policy level specifically targeted the structural drivers of health-related stigma among key or vulnerable populations. In another example from India, the Karnataka Health Promotion Trust organization educated female sex workers on their legal rights and implemented sensitization and awareness training with government officials, police, and journalists [53].

**Table 5:** Intervention-level descriptive statistics (*n* = 29)

	Total (%)
Region^a^	
Sub-Saharan Africa	18 (62%)
East Asia and Pacific	5 (17%)
South Asia	3 (10%)
Middle East and North Africa	2 (7%)
Europe and Central Asia	1 (3%)
Latin America and the Caribbean	1 (3%)
Associated health condition^a^	
HIV/AIDS	20 (69%)
Mental health	3 (10%)
Leprosy	2 (7%)
Sexually transmitted infections	2 (7%)
Tuberculosis	1 (3%)
Epilepsy	1 (3%)
Podoconiosis	1 (3%)
Lymphatic filariasis	1 (3%)
Intervention type^a^	
Information/Education	28 (97%)
Skills	13 (45%)
Counselling/Support	6 (21%)
Contact	6 (21%)
Structural	1 (3%)
Biomedical	1 (3%)
Target stigma domain^a^	
Driver	28 (97%)
Facilitator	4 (14%)
Manifestation	10 (34%)
Target stigma type^a^	
Community	24 (83%)
Experienced	11 (38%)
Internalized	9 (31%)
Anticipated	7 (24%)
Intervention level^a^	
Individual	23 (79%)
Interpersonal	14 (48%)
Organizational	6 (21%)
Community	11 (38%)
Public Policy	1 (3%)
TIDieR intervention specification	
Mean reporting score (SD)	60% (10%)
Why	28 (97%)
What materials	25 (86%)
What procedures	28 (97%)
Who provided	25 (86%)
How	29 (100%)
Where	27 (93%)
When and how much	26 (90%)
Tailoring	5 (17%)
Modifications	2 (7%)
How fidelity was assessed	4 (14%)
Level of fidelity	3 (10%)

^a^≥ 1 response per study possible

Adherence to the TIDieR checklist for reporting interventions was uneven. On average, interventions met 60% (SD 10%) of the TIDieR criteria. All interventions specified how they were delivered – whether face-to-face, remotely, individually, or in a group, and the majority offered a rationale to justify the intervention (28, 97%) and described the procedures involved in delivering intervention components (28, 97%). Few interventions (5, 17%) documented how they were tailored to different target groups or contexts, and only 2 (7%) described modifications that took place over the course of implementation.

## Discussion

We systematically reviewed implementation research conducted in support of stigma reduction interventions in LMICs. A broad, inclusive definition of implementation research was used, considering any studies that reported implementation outcomes while evaluating stigma reduction interventions. Few studies were found, with the majority of these evaluating interventions to reduce HIV-related stigma, taking place in sub-Saharan Africa, and evaluating pilot or once-off interventions. The interventions in the sample were diverse, adopting a variety of tactics to reduce stigma, though those that had been implemented at scale tended to incorporate mass media or target structural changes, rather than individual-level support or service delivery. Further, none took a trans-diagnostic approach seeking to reduce stigma associated with multiple health conditions.

A critical assessment of these studies suggested three key gaps in the literature. First, no study in the sample explicitly incorporated a conceptual framework for implementation research, evaluated implementation strategies using a type 2 or 3 hybrid study design, nor used validated measures of implementation outcomes. Second, most studies focused on intervention acceptability and feasibility, and few assessed adoption, appropriateness, cost, fidelity, penetration, or sustainability. Third, intervention descriptions were sparse and often lacked the key details necessary for the eventual replication and adoption of those interventions. These gaps were consistent across the different stigmatizing health conditions – coverage of robust methods for implementation research was not greater among studies of interventions targeting any particular condition.

Theoretical frameworks, validated measures, and rigorous methods support the generalizability and ultimately promote the utility of implementation research [78]. Implementation science is a rapidly growing field, though essentially all available frameworks and measures for implementation determinants and outcomes have been developed in high-income countries [25, 30, 79]. Frameworks like the Consolidated Framework for Implementation Research are increasingly popular and have produced actionable results to enhance implementation in high-resource settings [80–83], though they may need to be translated and adapted to support implementation of stigma reduction and other complex interventions in LMICs. Improvements to measurement could also promote the comparability of findings across future stigma implementation studies, accelerating knowledge production in the field and easing the translation of findings into practice [84]. Robust measures are increasingly available [25], including measures of acceptability, appropriateness, feasibility [85], and sustainability [86, 87], though there is a major need for continued development and validation to ensure these are relevant to stigma interventions and valid in LMIC settings. With such measures and frameworks in hand, LMIC-based stigma researchers could start to assess how patient-, provider-, facility-, and community-level characteristics predict implementation outcomes. Such studies would help determine, for example, the projected health sector cost of providing in-service stigma reduction training to clinicians, or the patient-level factors associated with preference for peer counselors over lay counselors. Subsequent type 2 and 3 effectiveness-implementation hybrid study designs could compare implementation strategies and observe changes in relevant outcomes [31], for example, experimenting with the counselor cadre and assessing relative levels of adoption. Of course, for all this to be feasible, capacity-building and funding for implementation science among stigma researchers in LMICs is critical. Few opportunities for training and support of LMIC-based implementation researchers are currently available [88].

Future research (Box 1) will need to assess the complete range of implementation outcomes to further strengthen the evidence base for the delivery and scale-up of effective stigma reduction interventions. Studies in this sample concentrated on assessing acceptability and feasibility and rarely measured other implementation outcomes. For example, only five studies measured provider- or facility-level adoption or penetration. As such, little is known about the factors associated with the uptake of stigma reduction interventions by health facilities, staff, patients, or communities in LMICs. Appropriateness, fidelity, cost, and sustainability were also seldom evaluated. Appropriateness is important because uptake of an intervention is unlikely unless community members, patients, and providers perceive its utility and compatibility with their other activities. One study used an innovative approach to improve the appropriateness of a stigma reduction intervention by involving community members with leprosy as staff members to inform study design and implementation [67]. Another asked community members to help select and tailor intervention components to address local concerns [61]. Fidelity has been shown to be critical to ensuring that effectiveness is maximized and successful outcomes are replicable across settings [89]. Evidence of cost and cost-effectiveness is necessary to justify scale-up and funding by health systems and donors. Finally, sustainability ensures investments into stigma reduction efforts are not wasted [90, 91].

Detailed, transparent descriptions of interventions in manuscripts and supplemental materials are also important to ensure others can replicate the work and achieve comparable results to those seen in effectiveness studies [92]. The majority of stigma interventions in the sample performed well against the TIDieR criteria, offering some description of the who, what, when, where, and why of intervention delivery [41], though descriptions were generally sparse, and few manuscripts offered links to formal manuals or protocols detailing intervention content and procedures. This is consistent with other reviews highlighting deficiencies in the comprehensive reporting of processes for complex interventions [93]. Moreover, few studies in the sample reported on intervention tailoring, modifications that were made over the course of the study, or fidelity assessment. Stigma is multi-dimensional; as a result, successful stigma interventions are complex, operating across multiple components and socio-ecological levels [15]. Complex interventions like these work best when peripheral components are tailored to local contexts [94]; it is therefore important to define the core, standardized parts of an intervention, and those that can be or have been adapted to suit local needs. As noted above, fidelity assessment is important to ensuring effectiveness; more frequent reporting of fidelity would serve both to increase the range of implementation outcomes assessed and to improve performance against the TIDieR criteria. Future stigma implementation research could ease the translation of findings into practice and deepen intervention specification by providing intervention materials as manuscript appendices, comprehensively documenting and reporting adaptations or modifications to interventions, and incorporating fidelity assessment into implementation and evaluation [95].

This review had several limitations. First, studies of interventions with stigma reduction as a secondary objective or incidental effect were excluded, though many interventions have immense potential to reduce health-related stigma even if stigma reduction is not their primary goal. For example, integration of services to address stigmatizing conditions into primary care and other platforms (e.g., primary mental health care [96] or prevention of vertical transmission of HIV as part of routine antenatal care [97]) may improve service delivery and patient health outcomes and de-stigmatize the associated condition. Evaluations of the implementation of these approaches exist (e.g., using interviews to assess acceptability and feasibility of vertical transmission prevention and antenatal service integration in Kenya [98]) but were not captured by this review. Second, studies conducted in high-income countries were excluded, though they may represent a significant proportion of stigma implementation research. This review focused on the unique challenge of studying the implementation of stigma-specific interventions in LMICs, where there is a large burden of unaddressed stigma as well as significant financial and logistic constraints to deliver such interventions. Third, this review was focused on implementation science, seeking to develop generalizable knowledge beyond the individual context under study. Therefore, unpublished and non-peer-reviewed studies were excluded. We recognize that barriers to publication in academic journals are greater for investigators in LMIC settings. To limit bias against non-English speaking investigators, we did not restrict our search on the basis of language. Finally, the assessment of implementation outcomes by studies in the sample was too sparse to draw strong conclusions about factors that promote or inhibit successful and sustained implementation at scale.

## Conclusion

Implementation science has the potential to support the development, delivery, and dissemination of stigma reduction interventions in LMICs, though usage to date has been limited. Rigorous stigma implementation research is urgently needed. There are clear barriers to successful implementation of stigma reduction interventions, especially in LMICs. Given these barriers, implementation science can help maximize the population health impact of stigma reduction interventions by allowing researchers to test and refine implementation strategies, develop new approaches to improve their interventions in various settings, explore and understand the causal mechanisms between intervention and impact, and generate evidence to convince policy-makers of the value of scale-up [99]. Such research will help us deliver on the promise of interventions to alleviate the burden of stigma worldwide.

**Box 1** Recommendations for future stigma implementation research • Incorporate theoretical frameworks for implementation research, validated measures of implementation outcomes, and hybrid study designs

• Assess how intervention-, implementation-, patient-, provider-, facility-, or community-level characteristics are associated with variation in implementation outcomes

• Assess the complete range of implementation outcomes, especially cost and sustainability

• Include detailed, transparent descriptions of interventions in manuscripts and supplemental materials

## Additional file

Additional file 1: **S1.** Systematic review search strategy. Search terms, number of results, and filters used when collecting studies from databases for the systematic review. **S2.** Abstraction form and dataset. Abstraction form and dataset used for our analysis. (ZIP 148 kb)

## Abbreviations

LMICs: low- and middle-income countries
TIDieR: Template for Intervention Description and Replication
SD: standard deviation
