# Supplementary material for: Implementation science and stigma reduction interventions in low- and middle-income countries: a systematic review
Source: BMC Med. 2019 Feb 15;17:6. doi: 10.1186/s12916-018-1237-x (PMC6376798; doi:10.1186/s12916-018-1237-x)
Supplement: Supplementary file 1 — S1. Systematic review search strategy. Search terms, number of results, and filters used when collecting studies from databases for the systematic review. S2. Abstraction form and dataset. Abstraction form and dataset used for our analysis. (ZIP 148 kb) [file 12916_2018_1237_MOESM1_ESM.zip › 20180531 Additional File 1 - Search StrategyR2.docx]

**Additional File 1: Search Strategy**

PubMed:

(social stigma[mh] OR shame[mh] OR prejudice[mh] OR "Discrimination (Psychology)"[mh] OR stigma[tw] OR stigmas[tw] OR stigmati*[tw] OR stereotyp*[tw] OR shame[tw] OR shames[tw] OR shaming[tw] OR discrimination[tw] OR discriminating[tw] OR “social distance”[tw] OR prejudice[tw] OR prejudices[tw] OR blame[tw] OR blames[tw])

AND

(intervention[tw] OR interventions[tw] OR intervene[tw] OR intervened[tw] OR intervening[tw] OR implement[tw] OR implements[tw] OR implemented[tw] OR implementation[tw] OR implementations[tw] OR program[tw] OR programs[tw] OR programming[tw] OR impact[tw] OR impacts[tw])

AND

(acceptability[tw] OR acceptable[tw] OR adoption[tw] OR adopted[tw] OR approved[tw] OR approval[tw] OR enacted[tw] OR endorsed[tw] OR endorsement[tw] OR maintained[tw] OR maintenance[tw] OR embraced[tw] OR selected[tw] OR selection[tw] OR chosen[tw] OR choice[tw] OR appropriateness[tw] OR appropriate[tw] OR suitable[tw] OR suitability[tw] OR suited[tw] OR apt[tw] OR useful[tw] OR usefulness[tw] OR fit[tw] OR feasible[tw] OR feasibility[tw] OR achievable[tw] OR advantageous[tw] OR attainable[tw] OR practicable[tw] OR practical[tw] OR viable[tw] OR workable[tw] OR worthwhile[tw] OR fidelity[tw] OR adherent[tw] OR adherence[tw] OR accuracy[tw] OR accurate[tw] OR precision[tw] OR precise[tw] OR uniform[tw] OR uniformity[tw] OR exact[tw] OR close[tw] OR cost[tw] OR economic*[tw] OR expense[tw] OR expensive[tw] OR expenditure[tw] OR expenditures[tw] OR pay[tw] OR paid[tw] OR price[tw] OR budget[tw] OR penetration[tw] OR penetrated[tw] OR diffusion[tw] OR sustainable[tw] OR sustained[tw] OR sustainability[tw] OR implementation science[tw] OR implementation research[tw] OR dissemination research[tw] OR “D&I”[tw] OR “D and I”[tw] OR implementation outcome[tw] OR implementation outcomes[tw])

AND

(Afghanistan[tw] OR Albania[tw] OR Algeria[tw] OR Samoa[tw] OR Angola[tw] OR Antigua[tw] OR Barbuda[tw] OR Argentina[tw] OR Armenia[tw] OR Azerbaijan[tw] OR Bangladesh[tw] OR Belarus[tw] OR Belize[tw] OR Benin[tw] OR Bhutan[tw] OR Bolivia[tw] OR Bosnia[tw] OR Herzegovina[tw] OR Botswana[tw] OR Brazil[tw] OR Bulgaria[tw] OR Burkina Faso[tw] OR Burundi[tw] OR Cambodia[tw] OR Cameroon[tw] OR Cabo Verde[tw] OR Central African Republic[tw] OR Chad[tw] OR Chile[tw] OR China[tw] OR Colombia[tw] OR Comoros[tw] OR Congo[tw] OR Costa Rica[tw] OR Côte d'Ivoire[tw] OR Cote d’Ivoire[tw] OR Ivory[tw] OR Cuba[tw] OR Djibouti[tw] OR Dominica[tw] OR Dominican[tw] OR Ecuador[tw] OR Egypt[tw] OR Salvador[tw] OR Eritrea[tw] OR Ethiopia[tw] OR Fiji[tw] OR Gabon[tw] OR Gambia[tw] OR Georgia[tw] OR Ghana[tw] OR Grenada[tw] OR Guatemala[tw] OR Guinea[tw] OR Guinea-Bissau[tw] OR Guyana[tw] OR Haiti[tw] OR Honduras[tw] OR India[tw] OR Indonesia[tw] OR Iran[tw] OR Iraq[tw] OR Jamaica[tw] OR Jordan[tw] OR Kazakhstan[tw] OR Kenya[tw] OR Kiribati[tw] OR Korea [tw] OR Kosovo[tw] OR Kyrgyz [tw] OR Lao[tw] OR Laos[tw] OR Latvia[tw] OR Lebanon[tw] OR Lesotho[tw] OR Liberia[tw] OR Libya[tw] OR Lithuania[tw] OR Macedonia[tw] OR Madagascar[tw] OR Malawi[tw] OR Malaysia[tw] OR Maldives[tw] OR Mali[tw] OR Marshall[tw] OR Mauritania[tw] OR Mauritius[tw] OR Mexico[tw] OR Micronesia[tw] OR Moldova[tw] OR Mongolia[tw] OR Montenegro[tw] OR Morocco[tw] OR Mozambique[tw] OR Myanmar[tw] OR Namibia[tw] OR Nepal[tw] OR Nicaragua[tw] OR Niger[tw] OR Nigeria[tw] OR Pakistan[tw] OR Palau[tw] OR Panama[tw] OR Papua New Guinea[tw] OR Paraguay[tw] OR Peru[tw] OR Philippines[tw] OR Romania[tw] OR Russia[tw] OR Russian[tw] OR Rwanda[tw] OR Samoa[tw] OR Sao Tome[tw] OR Senegal[tw] OR Serbia[tw] OR Seychelles[tw] OR Sierra Leone[tw] OR Solomon Islands[tw] OR Somalia[tw] OR South Africa[tw] OR Sri Lanka[tw] OR St. Lucia[tw] OR St. Vincent[tw] OR Grenadines[tw] OR Sudan[tw] OR Suriname[tw] OR Swaziland[tw] OR Syrian[tw] OR Syria[tw] OR Tajikistan[tw] OR Tanzania[tw] OR Thailand[tw] OR Timor-Leste[tw] OR Togo[tw] OR Tonga[tw] OR Tunisia[tw] OR Turkey[tw] OR Turkmenistan[tw] OR Tuvalu[tw] OR Uganda[tw] OR Ukraine[tw] OR Uruguay[tw] OR Uzbekistan[tw] OR Vanuatu[tw] OR Venezuela[tw] OR Vietnam[tw] OR “West Bank”[tw] OR Gaza[tw] OR Yemen[tw] OR Zambia[tw] OR Zimbabwe [tw] OR developing countries[mh] OR “developing countries”[tw] OR “resource-limited”[tw] OR “resource-constrained”[tw] OR “low- and middle-income”[tw] OR LMIC[tw] OR “third world”[tw] OR “low income countries”[tw])

PsycINFO:

(stigma OR stigmas OR stigmati* OR stereotyp* OR shame OR shames OR shaming OR discrimination OR discriminating OR “social distance” OR prejudice OR prejudices OR blame OR blames)

AND

(intervention OR interventions OR intervene OR intervened OR intervening OR implement OR implements OR implemented OR implementation OR implementations OR program OR programs OR programming OR impact OR impacts)

AND

(acceptability OR acceptable OR adoption OR adopted OR approved OR approval OR enacted OR endorsed OR endorsement OR maintained OR maintenance OR embraced OR selected OR selection OR chosen OR choice OR appropriateness OR appropriate OR suitable OR suitability OR suited OR apt OR useful OR usefulness OR fit OR feasible OR feasibility OR achievable OR advantageous OR attainable OR practicable OR practical OR viable OR workable OR worthwhile OR fidelity OR adherent OR adherence OR accuracy OR accurate OR precision OR precise OR uniform OR uniformity OR exact OR close OR cost OR economic* OR expense OR expensive OR expenditure OR expenditures OR pay OR paid OR price OR budget OR penetration OR penetrated OR diffusion OR sustainable OR sustained OR sustainability OR implementation science OR implementation research OR dissemination research OR “D&I” OR “D and I” OR implementation outcome OR implementation outcomes)

AND

(Afghanistan OR Albania OR Algeria OR Samoa OR Angola OR Antigua OR Barbuda OR Argentina OR Armenia OR Azerbaijan OR Bangladesh OR Belarus OR Belize OR Benin OR Bhutan OR Bolivia OR Bosnia OR Herzegovina OR Botswana OR Brazil OR Bulgaria OR Burkina Faso OR Burundi OR Cambodia OR Cameroon OR Cabo Verde OR Central African Republic OR Chad OR Chile OR China OR Colombia OR Comoros OR Congo OR Costa Rica OR Côte d'Ivoire OR Cote d’Ivoire OR Ivory OR Cuba OR Djibouti OR Dominica OR Dominican OR Ecuador OR Egypt OR Salvador OR Eritrea OR Ethiopia OR Fiji OR Gabon OR Gambia OR Georgia OR Ghana OR Grenada OR Guatemala OR Guinea OR Guinea-Bissau OR Guyana OR Haiti OR Honduras OR India OR Indonesia OR Iran OR Iraq OR Jamaica OR Jordan OR Kazakhstan OR Kenya OR Kiribati OR Korea OR Kosovo OR Kyrgyz OR Lao OR Laos OR Latvia OR Lebanon OR Lesotho OR Liberia OR Libya OR Lithuania OR Macedonia OR Madagascar OR Malawi OR Malaysia OR Maldives OR Mali OR Marshall OR Mauritania OR Mauritius OR Mexico OR Micronesia OR Moldova OR Mongolia OR Montenegro OR Morocco OR Mozambique OR Myanmar OR Namibia OR Nepal OR Nicaragua OR Niger OR Nigeria OR Pakistan OR Palau OR Panama OR Papua New Guinea OR Paraguay OR Peru OR Philippines OR Romania OR Russia OR Russian OR Rwanda OR Samoa OR Sao Tome OR Senegal OR Serbia OR Seychelles OR Sierra Leone OR Solomon Islands OR Somalia OR South Africa OR Sri Lanka OR St. Lucia OR St. Vincent OR Grenadines OR Sudan OR Suriname OR Swaziland OR Syrian OR Syria OR Tajikistan OR Tanzania OR Thailand OR Timor-Leste OR Togo OR Tonga OR Tunisia OR Turkey OR Turkmenistan OR Tuvalu OR Uganda OR Ukraine OR Uruguay OR Uzbekistan OR Vanuatu OR Venezuela OR Vietnam OR “West Bank” OR Gaza OR Yemen OR Zambia OR Zimbabwe OR “developing countries” OR “resource-limited” OR “resource-constrained” OR “low- and middle-income” OR LMIC OR “third world” OR “low income countries”)

CINAHL:

(stigma OR stigmas OR stigmati* OR stereotyp* OR shame OR shames OR shaming OR discrimination OR discriminating OR “social distance” OR prejudice OR prejudices OR blame OR blames)

AND

(intervention OR interventions OR intervene OR intervened OR intervening OR implement OR implements OR implemented OR implementation OR implementations OR program OR programs OR programming OR impact OR impacts)

AND

(acceptability OR acceptable OR adoption OR adopted OR approved OR approval OR enacted OR endorsed OR endorsement OR maintained OR maintenance OR embraced OR selected OR selection OR chosen OR choice OR appropriateness OR appropriate OR suitable OR suitability OR suited OR apt OR useful OR usefulness OR fit OR feasible OR feasibility OR achievable OR advantageous OR attainable OR practicable OR practical OR viable OR workable OR worthwhile OR fidelity OR adherent OR adherence OR accuracy OR accurate OR precision OR precise OR uniform OR uniformity OR exact OR close OR cost OR economic* OR expense OR expensive OR expenditure OR expenditures OR pay OR paid OR price OR budget OR penetration OR penetrated OR diffusion OR sustainable OR sustained OR sustainability OR implementation science OR implementation research OR dissemination research OR “D&I” OR “D and I” OR implementation outcome OR implementation outcomes)

AND

(Afghanistan OR Albania OR Algeria OR Samoa OR Angola OR Antigua OR Barbuda OR Argentina OR Armenia OR Azerbaijan OR Bangladesh OR Belarus OR Belize OR Benin OR Bhutan OR Bolivia OR Bosnia OR Herzegovina OR Botswana OR Brazil OR Bulgaria OR Burkina Faso OR Burundi OR Cambodia OR Cameroon OR Cabo Verde OR Central African Republic OR Chad OR Chile OR China OR Colombia OR Comoros OR Congo OR Costa Rica OR Côte d'Ivoire OR Cote d’Ivoire OR Ivory OR Cuba OR Djibouti OR Dominica OR Dominican OR Ecuador OR Egypt OR Salvador OR Eritrea OR Ethiopia OR Fiji OR Gabon OR Gambia OR Georgia OR Ghana OR Grenada OR Guatemala OR Guinea OR Guinea-Bissau OR Guyana OR Haiti OR Honduras OR India OR Indonesia OR Iran OR Iraq OR Jamaica OR Jordan OR Kazakhstan OR Kenya OR Kiribati OR Korea OR Kosovo OR Kyrgyz OR Lao OR Laos OR Latvia OR Lebanon OR Lesotho OR Liberia OR Libya OR Lithuania OR Macedonia OR Madagascar OR Malawi OR Malaysia OR Maldives OR Mali OR Marshall OR Mauritania OR Mauritius OR Mexico OR Micronesia OR Moldova OR Mongolia OR Montenegro OR Morocco OR Mozambique OR Myanmar OR Namibia OR Nepal OR Nicaragua OR Niger OR Nigeria OR Pakistan OR Palau OR Panama OR Papua New Guinea OR Paraguay OR Peru OR Philippines OR Romania OR Russia OR Russian OR Rwanda OR Samoa OR Sao Tome OR Senegal OR Serbia OR Seychelles OR Sierra Leone OR Solomon Islands OR Somalia OR South Africa OR Sri Lanka OR St. Lucia OR St. Vincent OR Grenadines OR Sudan OR Suriname OR Swaziland OR Syrian OR Syria OR Tajikistan OR Tanzania OR Thailand OR Timor-Leste OR Togo OR Tonga OR Tunisia OR Turkey OR Turkmenistan OR Tuvalu OR Uganda OR Ukraine OR Uruguay OR Uzbekistan OR Vanuatu OR Venezuela OR Vietnam OR “West Bank” OR Gaza OR Yemen OR Zambia OR Zimbabwe OR “developing countries” OR “resource-limited” OR “resource-constrained” OR “low- and middle-income” OR LMIC OR “third world” OR “low income countries”)

(stigma OR stigmas OR stigmati* OR stereotyp* OR shame OR shames OR shaming OR discrimination OR discriminating OR “social distance” OR prejudice OR prejudices OR blame OR blames)

AND

(intervention OR interventions OR intervene OR intervened OR intervening OR implement OR implements OR implemented OR implementation OR implementations OR program OR programs OR programming OR impact OR impacts)

AND

(acceptability OR acceptable OR adoption OR adopted OR approved OR approval OR enacted OR endorsed OR endorsement OR maintained OR maintenance OR embraced OR selected OR selection OR chosen OR choice OR appropriateness OR appropriate OR suitable OR suitability OR suited OR apt OR useful OR usefulness OR fit OR feasible OR feasibility OR achievable OR advantageous OR attainable OR practicable OR practical OR viable OR workable OR worthwhile OR fidelity OR adherent OR adherence OR accuracy OR accurate OR precision OR precise OR uniform OR uniformity OR exact OR close OR cost OR economic* OR expense OR expensive OR expenditure OR expenditures OR pay OR paid OR price OR budget OR penetration OR penetrated OR diffusion OR sustainable OR sustained OR sustainability OR “implementation science” OR “implementation research” OR “dissemination research” OR “D&I” OR “D and I” OR “implementation outcome” OR “implementation outcomes”)

AND

(Afghanistan OR Albania OR Algeria OR Samoa OR Angola OR Antigua OR Barbuda OR Argentina OR Armenia OR Azerbaijan OR Bangladesh OR Belarus OR Belize OR Benin OR Bhutan OR Bolivia OR Bosnia OR Herzegovina OR Botswana OR Brazil OR Bulgaria OR “Burkina Faso” OR Burundi OR Cambodia OR Cameroon OR “Cabo Verde” OR “Central African Republic” OR Chad OR Chile OR China OR Colombia OR Comoros OR Congo OR “Costa Rica” OR Ivoire OR Ivory OR Cuba OR Djibouti OR Dominica OR Dominican OR Ecuador OR Egypt OR Salvador OR Eritrea OR Ethiopia OR Fiji OR Gabon OR Gambia OR Georgia OR Ghana OR Grenada OR Guatemala OR Guinea OR “Guinea-Bissau” OR Guyana OR Haiti OR Honduras OR India OR Indonesia OR Iran OR Iraq OR Jamaica OR Jordan OR Kazakhstan OR Kenya OR Kiribati OR Korea OR Kosovo OR Kyrgyz OR Lao OR Laos OR Latvia OR Lebanon OR Lesotho OR Liberia OR Libya OR Lithuania OR Macedonia OR Madagascar OR Malawi OR Malaysia OR Maldives OR Mali OR Marshall OR Mauritania OR Mauritius OR Mexico OR Micronesia OR Moldova OR Mongolia OR Montenegro OR Morocco OR Mozambique OR Myanmar OR Namibia OR Nepal OR Nicaragua OR Niger OR Nigeria OR Pakistan OR Palau OR Panama OR “Papua New Guinea” OR Paraguay OR Peru OR Philippines OR Romania OR Russia OR Russian OR Rwanda OR Samoa OR “Sao Tome” OR Senegal OR Serbia OR Seychelles OR “Sierra Leone” OR “Solomon Islands” OR Somalia OR “South Africa” OR “Sri Lanka” OR “St. Lucia” OR “St. Vincent” OR Grenadines OR Sudan OR Suriname OR Swaziland OR Syrian OR Syria OR Tajikistan OR Tanzania OR Thailand OR “Timor-Leste” OR Togo OR Tonga OR Tunisia OR Turkey OR Turkmenistan OR Tuvalu OR Uganda OR Ukraine OR Uruguay OR Uzbekistan OR Vanuatu OR Venezuela OR Vietnam OR “West Bank” OR Gaza OR Yemen OR Zambia OR Zimbabwe OR “developing countries” OR “resource-limited” OR “resource-constrained” OR “low- and middle-income” OR LMIC OR “third world” OR “low income countries”)

EMBASE:

(stigma:ti,ab,tn,kw OR stigmas:ti,ab,tn,kw OR stigmati*:ti,ab,tn,kw OR stereotyp*:ti,ab,tn,kw OR shame:ti,ab,tn,kw OR shames:ti,ab,tn,kw OR shaming:ti,ab,tn,kw OR discrimination:ti,ab,tn,kw OR discriminating:ti,ab,tn,kw OR “social distance”:ti,ab,tn,kw OR prejudice:ti,ab,tn,kw OR prejudices:ti,ab,tn,kw OR blame:ti,ab,tn,kw OR blames:ti,ab,tn,kw)

AND

(intervention:ti,ab,tn,kw OR interventions:ti,ab,tn,kw OR intervene:ti,ab,tn,kw OR intervened:ti,ab,tn,kw OR intervening:ti,ab,tn,kw OR implement:ti,ab,tn,kw OR implements:ti,ab,tn,kw OR implemented:ti,ab,tn,kw OR implementation:ti,ab,tn,kw OR implementations:ti,ab,tn,kw OR program:ti,ab,tn,kw OR programs:ti,ab,tn,kw OR programming:ti,ab,tn,kw OR impact:ti,ab,tn,kw OR impacts:ti,ab,tn,kw)

AND

(acceptability:ti,ab,tn,kw OR acceptable:ti,ab,tn,kw OR adoption:ti,ab,tn,kw OR adopted:ti,ab,tn,kw OR approved:ti,ab,tn,kw OR approval:ti,ab,tn,kw OR enacted:ti,ab,tn,kw OR endorsed:ti,ab,tn,kw OR endorsement:ti,ab,tn,kw OR maintained:ti,ab,tn,kw OR maintenance:ti,ab,tn,kw OR embraced:ti,ab,tn,kw OR selected:ti,ab,tn,kw OR selection:ti,ab,tn,kw OR chosen:ti,ab,tn,kw OR choice:ti,ab,tn,kw OR appropriateness:ti,ab,tn,kw OR appropriate:ti,ab,tn,kw OR suitable:ti,ab,tn,kw OR suitability:ti,ab,tn,kw OR suited:ti,ab,tn,kw OR apt:ti,ab,tn,kw OR useful:ti,ab,tn,kw OR usefulness:ti,ab,tn,kw OR fit:ti,ab,tn,kw OR feasible:ti,ab,tn,kw OR feasibility:ti,ab,tn,kw OR achievable:ti,ab,tn,kw OR advantageous:ti,ab,tn,kw OR attainable:ti,ab,tn,kw OR practicable:ti,ab,tn,kw OR practical:ti,ab,tn,kw OR viable:ti,ab,tn,kw OR workable:ti,ab,tn,kw OR worthwhile:ti,ab,tn,kw OR fidelity:ti,ab,tn,kw OR adherent:ti,ab,tn,kw OR adherence:ti,ab,tn,kw OR accuracy:ti,ab,tn,kw OR accurate:ti,ab,tn,kw OR precision:ti,ab,tn,kw OR precise:ti,ab,tn,kw OR uniform:ti,ab,tn,kw OR uniformity:ti,ab,tn,kw OR exact:ti,ab,tn,kw OR close:ti,ab,tn,kw OR cost:ti,ab,tn,kw OR economic*:ti,ab,tn,kw OR expense:ti,ab,tn,kw OR expensive:ti,ab,tn,kw OR expenditure:ti,ab,tn,kw OR expenditures:ti,ab,tn,kw OR pay:ti,ab,tn,kw OR paid:ti,ab,tn,kw OR price:ti,ab,tn,kw OR budget:ti,ab,tn,kw OR penetration:ti,ab,tn,kw OR penetrated:ti,ab,tn,kw OR diffusion:ti,ab,tn,kw OR sustainable:ti,ab,tn,kw OR sustained:ti,ab,tn,kw OR sustainability:ti,ab,tn,kw OR “implementation science”:ti,ab,tn,kw OR “implementation research”:ti,ab,tn,kw OR “dissemination research”:ti,ab,tn,kw OR “D&I”:ti,ab,tn,kw OR “D and I”:ti,ab,tn,kw OR “implementation outcome”:ti,ab,tn,kw OR “implementation outcomes”:ti,ab,tn,kw)

AND

(Afghanistan:ti,ab,tn,kw OR Albania:ti,ab,tn,kw OR Algeria:ti,ab,tn,kw OR Samoa:ti,ab,tn,kw OR Angola:ti,ab,tn,kw OR Antigua:ti,ab,tn,kw OR Barbuda:ti,ab,tn,kw OR Argentina:ti,ab,tn,kw OR Armenia:ti,ab,tn,kw OR Azerbaijan:ti,ab,tn,kw OR Bangladesh:ti,ab,tn,kw OR Belarus:ti,ab,tn,kw OR Belize:ti,ab,tn,kw OR Benin:ti,ab,tn,kw OR Bhutan:ti,ab,tn,kw OR Bolivia:ti,ab,tn,kw OR Bosnia:ti,ab,tn,kw OR Herzegovina:ti,ab,tn,kw OR Botswana:ti,ab,tn,kw OR Brazil:ti,ab,tn,kw OR Bulgaria:ti,ab,tn,kw OR “Burkina Faso”:ti,ab,tn,kw OR Burundi:ti,ab,tn,kw OR Cambodia:ti,ab,tn,kw OR Cameroon:ti,ab,tn,kw OR “Cabo Verde”:ti,ab,tn,kw OR “Central African Republic”:ti,ab,tn,kw OR Chad:ti,ab,tn,kw OR Chile:ti,ab,tn,kw OR China:ti,ab,tn,kw OR Colombia:ti,ab,tn,kw OR Comoros:ti,ab,tn,kw OR Congo:ti,ab,tn,kw OR “Costa Rica”:ti,ab,tn,kw OR Ivoire:ti,ab,tn,kw OR Ivory:ti,ab,tn,kw OR Cuba:ti,ab,tn,kw OR Djibouti:ti,ab,tn,kw OR Dominica:ti,ab,tn,kw OR Dominican:ti,ab,tn,kw OR Ecuador:ti,ab,tn,kw OR Egypt:ti,ab,tn,kw OR Salvador:ti,ab,tn,kw OR Eritrea:ti,ab,tn,kw OR Ethiopia:ti,ab,tn,kw OR Fiji:ti,ab,tn,kw OR Gabon:ti,ab,tn,kw OR Gambia:ti,ab,tn,kw OR Georgia:ti,ab,tn,kw OR Ghana:ti,ab,tn,kw OR Grenada:ti,ab,tn,kw OR Guatemala:ti,ab,tn,kw OR Guinea:ti,ab,tn,kw OR “Guinea-Bissau”:ti,ab,tn,kw OR Guyana:ti,ab,tn,kw OR Haiti:ti,ab,tn,kw OR Honduras:ti,ab,tn,kw OR India:ti,ab,tn,kw OR Indonesia:ti,ab,tn,kw OR Iran:ti,ab,tn,kw OR Iraq:ti,ab,tn,kw OR Jamaica:ti,ab,tn,kw OR Jordan:ti,ab,tn,kw OR Kazakhstan:ti,ab,tn,kw OR Kenya:ti,ab,tn,kw OR Kiribati:ti,ab,tn,kw OR Korea:ti,ab,tn,kw OR Kosovo:ti,ab,tn,kw OR Kyrgyz:ti,ab,tn,kw OR Lao:ti,ab,tn,kw OR Laos:ti,ab,tn,kw OR Latvia:ti,ab,tn,kw OR Lebanon:ti,ab,tn,kw OR Lesotho:ti,ab,tn,kw OR Liberia:ti,ab,tn,kw OR Libya:ti,ab,tn,kw OR Lithuania:ti,ab,tn,kw OR Macedonia:ti,ab,tn,kw OR Madagascar:ti,ab,tn,kw OR Malawi:ti,ab,tn,kw OR Malaysia:ti,ab,tn,kw OR Maldives:ti,ab,tn,kw OR Mali:ti,ab,tn,kw OR Marshall:ti,ab,tn,kw OR Mauritania:ti,ab,tn,kw OR Mauritius:ti,ab,tn,kw OR Mexico:ti,ab,tn,kw OR Micronesia:ti,ab,tn,kw OR Moldova:ti,ab,tn,kw OR Mongolia:ti,ab,tn,kw OR Montenegro:ti,ab,tn,kw OR Morocco:ti,ab,tn,kw OR Mozambique:ti,ab,tn,kw OR Myanmar:ti,ab,tn,kw OR Namibia:ti,ab,tn,kw OR Nepal:ti,ab,tn,kw OR Nicaragua:ti,ab,tn,kw OR Niger:ti,ab,tn,kw OR Nigeria:ti,ab,tn,kw OR Pakistan:ti,ab,tn,kw OR Palau:ti,ab,tn,kw OR Panama:ti,ab,tn,kw OR “Papua New Guinea”:ti,ab,tn,kw OR Paraguay:ti,ab,tn,kw OR Peru:ti,ab,tn,kw OR Philippines:ti,ab,tn,kw OR Romania:ti,ab,tn,kw OR Russia:ti,ab,tn,kw OR Russian:ti,ab,tn,kw OR Rwanda:ti,ab,tn,kw OR Samoa:ti,ab,tn,kw OR “Sao Tome”:ti,ab,tn,kw OR Senegal:ti,ab,tn,kw OR Serbia:ti,ab,tn,kw OR Seychelles:ti,ab,tn,kw OR “Sierra Leone”:ti,ab,tn,kw OR “Solomon Islands”:ti,ab,tn,kw OR Somalia:ti,ab,tn,kw OR “South Africa”:ti,ab,tn,kw OR “Sri Lanka”:ti,ab,tn,kw OR “St. Lucia”:ti,ab,tn,kw OR “St. Vincent”:ti,ab,tn,kw OR Grenadines:ti,ab,tn,kw OR Sudan:ti,ab,tn,kw OR Suriname:ti,ab,tn,kw OR Swaziland:ti,ab,tn,kw OR Syrian:ti,ab,tn,kw OR Syria:ti,ab,tn,kw OR Tajikistan:ti,ab,tn,kw OR Tanzania:ti,ab,tn,kw OR Thailand:ti,ab,tn,kw OR “Timor-Leste”:ti,ab,tn,kw OR Togo:ti,ab,tn,kw OR Tonga:ti,ab,tn,kw OR Tunisia:ti,ab,tn,kw OR Turkey:ti,ab,tn,kw OR Turkmenistan:ti,ab,tn,kw OR Tuvalu:ti,ab,tn,kw OR Uganda:ti,ab,tn,kw OR Ukraine:ti,ab,tn,kw OR Uruguay:ti,ab,tn,kw OR Uzbekistan:ti,ab,tn,kw OR Vanuatu:ti,ab,tn,kw OR Venezuela:ti,ab,tn,kw OR Vietnam:ti,ab,tn,kw OR “West Bank”:ti,ab,tn,kw OR Gaza:ti,ab,tn,kw OR Yemen:ti,ab,tn,kw OR Zambia:ti,ab,tn,kw OR Zimbabwe:ti,ab,tn,kw OR “developing countries”:ti,ab,tn,kw OR “resource-limited”:ti,ab,tn,kw OR “resource-constrained”:ti,ab,tn,kw OR “low- and middle-income”:ti,ab,tn,kw OR LMIC:ti,ab,tn,kw OR “third world”:ti,ab,tn,kw OR “low income countries”:ti,ab,tn,kw)

*Search Results* (15 November 2017):

| **Database** | **Number of results** | **Notes/Exclusions** |
| --- | --- | --- |
| PubMed | 3031 (3017 after import) | None. |
| PsycINFO | 2489 (1580 after import) | Exclude dissertations. |
| CINAHL | 1154 (364 after import) | Academic journals only. |
| EMBASE | 2651 (965 after import) | None. |
